# Incremental Predictive Value of Serum AST-to-ALT Ratio for Incident Metabolic Syndrome: The ARIRANG Study

**DOI:** 10.1371/journal.pone.0161304

**Published:** 2016-08-25

**Authors:** Dhananjay Yadav, Eunhee Choi, Song Vogue Ahn, Soon Koo Baik, Youn zoo Cho, Sang Baek Koh, Ji Hye Huh, Yoosoo Chang, Ki-Chul Sung, Jang Young Kim

**Affiliations:** 1 Department of Preventive Medicine, Yonsei University, Wonju College of Medicine, Wonju, Korea; 2 Institute of Lifestyle Medicine, Yonsei University, Wonju College of Medicine, Wonju, Korea; 3 Institute of Genomic Cohort, Yonsei University, Wonju College of Medicine, Wonju, Korea; 4 Department of Internal Medicine, Yonsei University, Wonju College of Medicine, Wonju, Korea; 5 Department of Cardiology, Yonsei University, Wonju College of Medicine, Wonju, Korea; 6 Department of Occupational and Environmental Medicine, Kangbuk Samsung Hospital, Sungkyunkwan University, School of Medicine, Seoul, Korea; 7 Division of Cardiology, Department of Medicine, Kangbuk Samsumg Hospital, Sungkyunkwan University School of Medicine, Seoul, Korea; Chang Gung Memorial Hospital Kaohsiung Branch, TAIWAN

## Abstract

**Aims:**

The ratio of aspartate aminotransferase (AST) to alanine aminotransferase (ALT) is of great interest as a possible novel marker of metabolic syndrome. However, longitudinal studies emphasizing the incremental predictive value of the AST-to-ALT ratio in diagnosing individuals at higher risk of developing metabolic syndrome are very scarce. Therefore, our study aimed to evaluate the AST-to-ALT ratio as an incremental predictor of new onset metabolic syndrome in a population-based cohort study.

**Material and Methods:**

The population-based cohort study included 2276 adults (903 men and 1373 women) aged 40–70 years, who participated from 2005–2008 (baseline) without metabolic syndrome and were followed up from 2008–2011. Metabolic syndrome was defined according to the harmonized definition of metabolic syndrome. Serum concentrations of AST and ALT were determined by enzymatic methods.

**Results:**

During an average follow-up period of 2.6-years, 395 individuals (17.4%) developed metabolic syndrome. In a multivariable adjusted model, the odds ratio (95% confidence interval) for new onset of metabolic syndrome, comparing the fourth quartile to the first quartile of the AST-to-ALT ratio, was 0.598 (0.422–0.853). The AST-to-ALT ratio also improved the area under the receiver operating characteristic curve (AUC) for predicting new cases of metabolic syndrome (0.715 vs. 0.732, P = 0.004). The net reclassification improvement of prediction models including the AST-to-ALT ratio was 0.23 (95% CI: 0.124–0.337, P<0.001), and the integrated discrimination improvement was 0.0094 (95% CI: 0.0046–0.0143, P<0.001).

**Conclusions:**

The AST-to-ALT ratio independently predicted the future development of metabolic syndrome and had incremental predictive value for incident metabolic syndrome.

## Introduction

Metabolic syndrome is a constellation of several cardiovascular risk factors, including hyperglycemia, obesity, high blood pressure, hypertriglyceridemia, and low HDL cholesterol [1]. It is well reported that metabolic syndrome escalates the risk of type 2 diabetes and cardiovascular disease [2–4]. The deadly consequences and elevated prevalence of metabolic syndrome motivates interest in understanding the causes and risk factors in population-based cohort studies. Besides the well-accepted metabolic syndrome components, diverse risk factors also have been recognized as non-traditional components, such as hyperuricemia, microalbuminuria, and non-alcoholic fatty liver disease (NAFLD) [5–7].

A reduced ratio of aspartate aminotransferase (AST) to alanine aminotransferase (ALT) might be a surrogate measure of NAFLD, and is considered to be another aspect of hyperinsulinemia and insulin resistance [6, 8]. An increased AST-to-ALT ratio is strongly suggestive of alcoholic liver disease, whereas a reduced ratio specifies non-alcoholic steatohepatitis [9]. Recent studies have reported that an increased AST-to-ALT ratio is inversely associated with the future development of metabolic syndrome [10, 11]. However, the clinical utility and ability of the AST-to-ALT ratio in predicting metabolic syndrome and its individual components is not well understood. It is also speculated that this ratio could be used as a predictor of incident metabolic syndrome beyond the information contributed by each component of metabolic syndrome among healthy subjects. We hypothesized that a higher AST-to-ALT ratio would serve as a negative predictor of developing metabolic syndrome.

Hence, we studied the prospective association of the AST-to-ALT ratio with the risk of new-onset of metabolic syndrome and its individual components, as well as the discrimination value of the AST-to-ALT ratio in identifying participants who are likely to develop metabolic syndrome in the future. In addition, we calculated net reclassification improvement (NRI) and integrated discrimination index (IDI) to examine the incremental predictive value of the AST-to-ALT ratio as a novel biomarker for predicting future metabolic syndrome.

## Material and Methods

### Study population

Our study data were collected from the Korean Genome and Epidemiology Study (KoGES), an ongoing multicenter prospective cohort study designed to estimate the prevalence, incidence, and risk factors related with many disorders such as diabetes, hypertension, and cardiovascular disease [12–14]. The study enrolled adults in the rural region of Wonju and Pyeongchang in South Korea.

**Fig 1:**
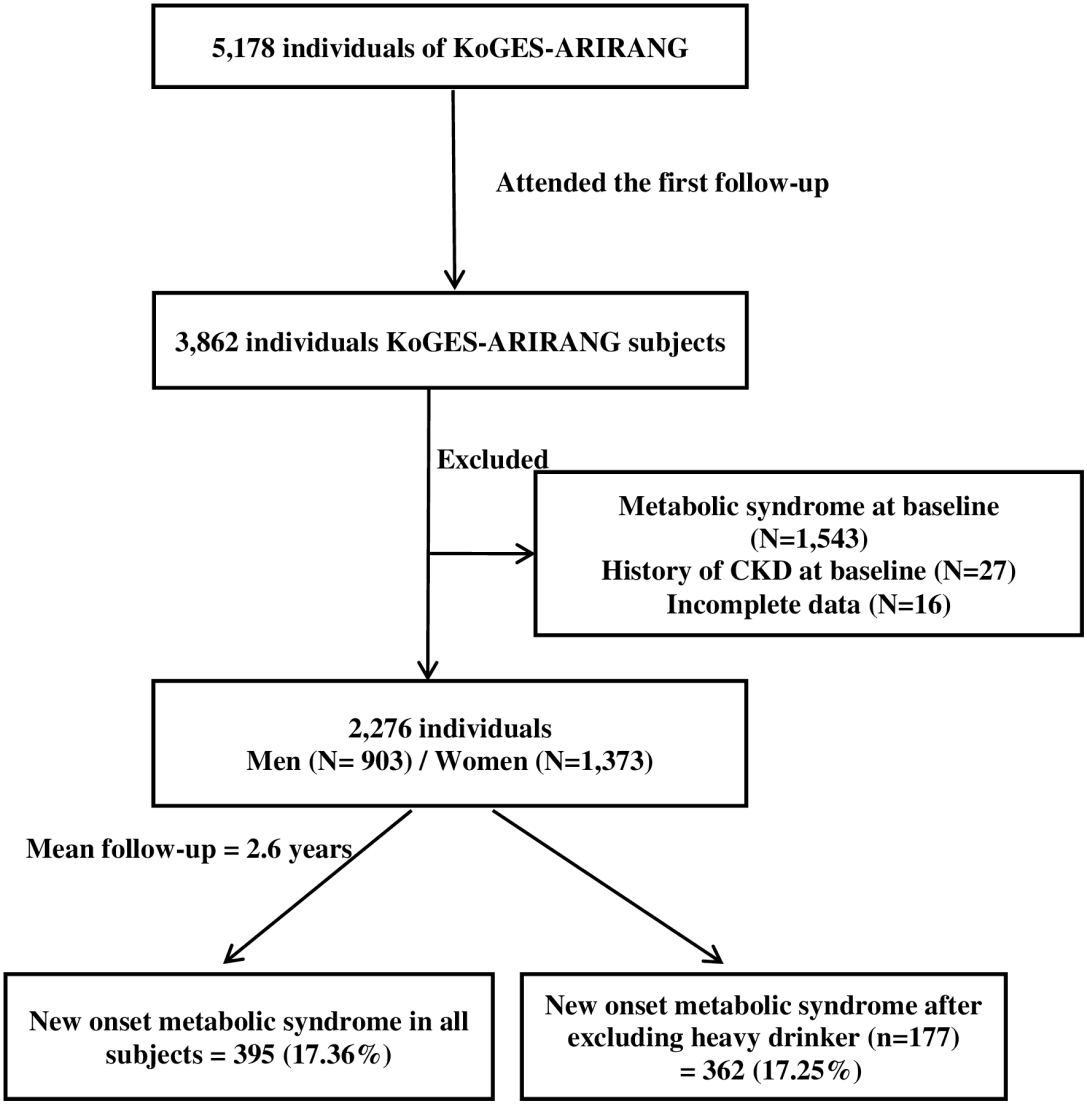
Description of the study population.

The baseline period of this study was from November 2005 to January 2008, and involved 5178 adults (2127 men and 3051 women) aged 40–70 years. The first follow-up study was carried out from April 2008–January 2011 and 3862 (74.6%) participants attended (mean time between appointments = 2.6 years). Participants with missing data (n = 16) and diagnosed with metabolic syndrome at baseline (n = 1543) were excluded. We also excluded 27 subjects with a history of cardiovascular disease at baseline. A total of 2276 individuals were involved in the current analysis (903 men and 1373 women) (Fig 1).

### Ethics statement

Written consent was obtained from each participant before the commencement of the study. The protocol was approved by the institutional review board (IRB; CR105024-026) of Wonju Severance Christian Hospital.

### Data collection and measurements

The study participants completed a health examination including the lifestyle questionnaire, and a medical history. Body weight and height were measured, with the participants barefooted and lightly dressed, and from these measures body mass index (BMI) was calculated. Waist circumference was measured using tape (SECA-200; SECA, Hamburg, Germany). Blood pressure was monitored from the right arm using a mercury sphygmomanometer (Baumanometer, Copiague, NY, USA) after the participant had rested for at least 5 min in a quiet room. Two consecutive measurements of systolic and diastolic pressure were taken at an interval of at least 5 min, and the average of the readings was used. The data for baseline information on smoking status and current alcohol intake was collected using a self-reported questionnaire (yes/no). The heavy drinker group was defined as those whose alcohol consumption exceeded 30 g/day [15].

After fasting for more than 12 h or overnight, venous blood samples were collected from each participant. Blood glucose, serum HDL-cholesterol, triglyceride, LDL-cholesterol (LDL-C), very low density lipoprotein (VLDL) cholesterol, aspartate transaminase (AST), and alanine transaminase (ALT) were measured by enzymatic methods (ADVIA 1650, Siemens, Tarrytown, NY, USA). High-sensitivity C-reactive protein (hs-CRP) was measured using the Denka Seiken (Tokyo, Japan) assay. Fasting insulin and homeostasis model assessment of insulin resistance (HOMA-IR) were also measured [16].

### Definition of metabolic syndrome

The end-point of the study was new onset of metabolic syndrome at the follow-up. The harmonized definition was used to diagnose metabolic syndrome in the participants [17, 18].

### Statistical methods

Data in this study are expressed as frequencies with percentage, means with standard deviation, or medians with interquartile range. It has been noted that the ratio of AST-to-ALT is used to classify fatty liver disease as alcoholic or non-alcoholic. In our study, we also analyzed the data with the exclusion of heavy drinkers (n = 177) to reveal the association between heavy drinking and liver injury. AST-to-ALT ratio was evaluated as a prediction tool for different components of metabolic syndrome over 2.6 years of follow-up by logistic regression. The odds ratio with 95% confidence intervals (95% CI) was estimated according to increasing quartiles of the AST-to-ALT ratio. The independent association between the baseline AST-to-ALT ratio and the onset of metabolic syndrome was analyzed by multivariate logistic regression. We utilized three models for the adjustment. First, we executed an age-related analysis. Second, we adjusted for age, BMI, LDL cholesterol, regular exercise, and smoking. Third, we adjusted for the factors in the second analysis with the addition of hs-CRP levels and HOMA-IR.

To calculate the added discrimination or incremental effect contributed by the AST-to-ALT ratio to predict future participants diagnosed with metabolic syndrome beyond the information furnished by the different components of the metabolic syndrome, our study analyzed the areas under the receiver operating characteristic curve (AUC) in models that contained HDL-cholesterol, waist circumference, systolic and diastolic blood pressure, triglycerides, and fasting glucose, with and without the AST-to-ALT ratio. Additionally, we calculated the category-free NRI and IDI for models with and without the AST-to-ALT ratio, to measure the improvement of corrected reclassification and sensitivity based on the addition of the serum AST-to-ALT ratio to the logistic model. NRI represents the difference between the proportion of participants moving up and the proportion of participants moving down for the development of metabolic syndrome, as well as the corresponding difference in proportions for those who did not have metabolic syndrome, by obtaining the difference of these two differences [19]. IDI represents the difference between the integrated difference in sensitivity and the integrated difference in one minus specificity for models with and without the new biomarker [20]. A *P*-value less than 0.05 was considered statistically significant, and all statistical analyses were accomplished using SAS, version 9.2 (SAS Institute, Cary, NC, USA).

## Results

### Patient characteristics

After an average follow-up of 2.6 years, 395 (17.4%) participants developed metabolic syndrome. The incidence of metabolic syndrome was similar after excluding heavy drinkers from the study. Baseline characteristics of the study subjects, categorized according to whether or not they had metabolic syndrome at follow-up, are shown in Table 1. Subjects who developed metabolic syndrome at follow-up were older and had significantly higher waist circumference, BMI, fasting blood glucose, blood pressure, LDL cholesterol, triglycerides, and HOMA-IR values than the non-metabolic syndrome participants.

**Table 1:** Baseline characteristics of the study subjects stratified by incident metabolic syndrome, and evaluation of the AST-to-ALT ratio in relation to the number of metabolic parameters used to define metabolic syndrome at follow-up.

Variables		All subjects			Excluding heavy drinkers		
		Metabolic syndrome (n = 395)	Non-metabolic syndrome (n = 1881)	p-value	Metabolic syndrome (n = 362)	Non-metabolic syndrome (n = 1737)	p-value
Age (y)		55.40 ± 7.89	53.91 ± 8.24	0.001	55.34 ± 7.87	53.88 ± 8.2	0.0019
Gender	Men	164 (18.16%)	739 (81.84%)	0.41	135 (18.05%)	613 (81.95%)	0.469
	Women	231 (16.82%)	1142 (83.18%)		227 (16.8%)	1124 (83.2%)	
Waist circumference (cm)		83.93 ± 7.0	79.28 ± 7.23	<0.0001	83.92 ± 7.04	78.98 ± 7.72	<0.0001
BMI (kg/m^2^)		24.99 ± 2.53	23.21 ± 2.66	<0.0001	25.08 ± 2.56	23.21 ± 2.68	<0.0001
Fasting glucose (mg/dL)		95.03 ± 18.39	90.3 ± 12.6	<0.0001	94.92 ± 18.92	90.08 ± 12.78	<0.0001
SBP (mmHg)		131.9 ± 18.52	124.2 ± 16.61	<0.0001	132 ± 18.46	123.9 ± 16.55	<0.0001
DBP (mmHg)		82.82 ± 11.35	79.44 ± 11.21	<0.0001	82.75 ± 11.27	79.2 ± 11.15	<0.0001
HDL cholesterol (mg/dL)		45.95 ± 9.9	50.06 ± 11.09	<0.0001	45.76 ± 9.79	49.74 ± 10.66	<0.0001
LDL cholesterol (mg/dL)		121 ± 30.33	114.6 ± 30.62	0.0002	122.4 ± 29.27	115 ± 30.78	<0.0001
Triglyceride (mg/dL)		132.7 ± 73.49	106.2 ± 54.79	<0.0001	127.5 ± 61.7	104.2 ± 52.57	<0.0001
hs-CRP (mg/L)		2.13 ± 5.66	1.75 ± 5.04	0.228	2.17 ± 5.84	1.76 ± 5.15	0.2204
HOMA-IR		1.95 ± 0.99	1.69 ± 0.96	<0.0001	1.98 ± 1.02	1.7 ± 0.96	<0.0001
AST (units/L)		28.07 ± 15.89	26.79 ± 14.09	0.1376	27.35 ± 15.7	26.14 ± 11.79	0.1662
ALT (units/L)		22.31 ± 14.97	26.34 ± 17.86	<0.0001	25.72 ± 17.45	21.75 ± 13.6	<0.0001
AST/ALT ratio		1.18 ± 0.35	1.32 ± 0.43	<0.0001	1.18 ± 0.36	1.32 ± 0.43	<0.0001
Smoker (%)		127 (32.23%)	511 (27.52%)	0.0456	104 (28.81%)	415 (23.97%)	0.0531
Exercise (%)		134 (33.92%)	573 (30.59%)	0.1939	122 (33.7%)	529 (30.6%)	0.2458
Drinking (%)		187 (47.46%)	821 (43.72%)	0.1737	154 (42.66%)	677 (39.04)	0.201
No. of components		AST/ALT ratio			AST/ALT ratio		
0		1.38 ± 0.48		<0.0001	1.38 ± 0.49		<0.0001
1		1.33 ± 0.43			1.34 ± 0.44		
2		1.26 ± 0.37			1.27 ± 0.37		
3		1.19 ± 0.37			1.2 ± 0.37		
≥4		1.14 ± 0.3			1.13 ± 0.29		

Data are expressed as mean ± standard deviation or number (%). BMI, Body mass index; SBP, Systolic blood pressure; DBP, Diastolic blood pressure; hs-CRP, high-sensitivity C-reactive protein; HOMA-IR, homeostasis model assessment-estimated insulin resistance; AST, aspartate aminotransferase; ALT, alanine aminotransferase

HDL-cholesterol and the AST-to-ALT ratio were significantly lower in participants who developed metabolic syndrome than in those who did not (all P<0.0001). The prevalence of smoking was significantly higher in the metabolic syndrome group (P<0.05), while there were no significant differences in regular exercise or current alcohol intake between the two groups. The estimates were similar when we excluded heavy drinkers from our analysis, except for smoking status, which nevertheless was found to not be significant. The elevated level of hs-CRP did not show any statistically significant difference between the metabolic syndrome group and the non-metabolic syndrome group. The AST-to-ALT ratio at baseline constantly decreased with the number of metabolic syndrome components developed in study subjects over the 2.6 years of follow-up (*P* for trend <0.0001). Moreover, we analyzed the data in a cross-sectional manner and the data corroborated the results of the prospective study analysis after 2.6 years of follow-up (Table A and B in S1 Tables).

### Multivariate Analysis for Predicting the Risk of Metabolic Syndrome According to Quartile Increment of AST-to-ALT ratio

We divided the study population into quartiles of AST-to-ALT ratios with cut-offs of 1.04, 1.25, and 1.5, and evaluated the association between baseline AST-to-ALT ratio and incident metabolic syndrome and its individual components at the follow-up visit. The incidence of metabolic syndrome in the first quartile of the AST-to-ALT ratio was 25.87%, which was reduced significantly to 18.68%, 12.5%, and 12.2% in the successive quartiles (Table 2). The crude odds ratio for the development of metabolic syndrome, comparing individuals in the fourth quartile with those in the first quartile of the AST-to-ALT ratio, was 0.398 (0.288–0.551, P for trend <0.0001). When the highest quartile of the AST-to-ALT ratio was compared with the first quartile, the age-adjusted odds ratio (95% CI) of metabolic syndrome was 0.364 (0.262–0.505) in all subjects and 0.367 (0.261–0.517) after excluding heavy drinkers. In the multivariate-adjusted model (Table 2), when the highest quartile of the AST-to-ALT ratio was compared to the lowest quartile, the odds ratio for the onset of metabolic syndrome was 0.598 (0.420–0.853, P = 0.0004). The analysis was repeated with the exclusion of heavy drinkers, as shown in Table 2, and the results were consistent. We calculated the odds ratio and 95% CI for incident metabolic syndrome based on the serial change of AST-to -ALT ratio. After adjustment for several confounding factors (i.e., model 3), the odds ratio for incident metabolic syndrome in the highest quartile of serial change of AST-to-ALT ratio compared with the lowest quartile of change in AST-to-ALT ratio was 0.796 (0.570–1.113), P = 0.1751. The study did not observe any significant difference for onset of metabolic syndrome stratified by the serial changes of AST-to-ALT ratio in all participants as well as after excluding heavy drinkers (Table C in S1 Tables). The study also analyzed the odds ratio for incident metabolic syndrome based on the quartiles of ALT and GGT levels (Table D in S1 Tables).

**Table 2:** Odds ratio and 95% confidence interval (CI) for new-onset metabolic syndrome according to different quartiles of AST-to-ALT ratios.

	Q1	Q2	Q3	Q4	p-value
All subjects	N = 576	N = 562	N = 630	N = 508	
AST-to-ALT ratio	~ 1.04	1.04–1.25	1.25–1.5	1.5 ~	
Incidence	149 (25.87%)	105 (18.68%)	79 (12.54%)	62 (12.2%)	<0.0001
Crude OR	1	0.658 (0.496–0.873)	0.411 (0.304–0.555)	0.398 (0.288–0.551)	<0.0001
Model 1	1	0.633 (0.476–0.842)	0.385 (0.284–0.522)	0.364 (0.262–0.505)	<0.0001
Model 2	1	0.755 (0.56–1.018)	0.505 (0.367–0.694)	0.571 (0.403–0.81)	0.0001
Model 3	1	0.778 (0.575–1.051)	0.522 (0.378–0.719)	0.598 (0.42–0.853)	0.0004
Excluding heavy drinkers	N = 527	N = 528	N = 578	N = 466	
AST-to-ALT ratio	~ 1.04	1.04–1.26	1.26–1.5	1.5 ~	
Incidence	137 (26%)	99 (18.75%)	69 (11.94%)	57 (12.23%)	<0.0001
Crude OR	1	0.657 (0.49–0.88)	0.386 (0.281–0.53)	0.397 (0.283–0.557)	<0.0001
Model 1	1	0.632 (0.47–0.848)	0.365 (0.265–0.503)	0.367 (0.261–0.517)	<0.0001
Model 2	1	0.759 (0.556–1.035)	0.494 (0.352–0.692)	0.595 (0.413–0.857)	0.0003
Model 3	1	0.786 (0.574–1.075)	0.513 (0.365–0.721)	0.627 (0.434–0.905)	0.0011

Data are OR (95% CI) or n (%). Model 1: adjusted for age. Model 2: Model 1, plus additional adjustment for baseline BMI, LDL cholesterol, smoking, regular exercise, and alcohol drinking. Model 3: Model 2, plus additional adjustment for baseline hs-CRP and HOMA-IR

### Odds Ratio for Each Component of Metabolic Syndrome Classified by the Baseline AST-to-ALT ratio

Table 3 displays the odds ratios for each component of metabolic syndrome, stratified by the baseline AST-to-ALT ratio. Subjects in the lowest AST-to-ALT ratio quartile had significantly higher odds for abnormal components of metabolic syndrome than those in the other three quartiles. Upon comparison with subjects in the first quartile of the AST-to-ALT ratio, subjects in the fourth quartile had significantly lower odds ratios for metabolic abnormalities, i.e., waist circumference, blood pressure, triglycerides, and blood glucose, in spite of not having any significant differences in HDL cholesterol. The crude odds ratios (95% CI) for large waist circumference, high triglycerides, low HDL cholesterol, elevated blood pressure, and elevated blood glucose were 0.331 (0.25–0.44), 0.450 (0.332–0.611), 0.889 (0.7–1.129), 0.814 (0.631–1.05), and 0.618 (0.451–0.847), respectively. Similar results were obtained after repeating the logistic analysis for subjects who were heavy alcohol drinkers.

**Table 3:** Odds ratios for individual components of metabolic syndrome according to baseline AST-to-ALT ratio.

		Q1	Q2	Q3	Q4	p-value
All subjects		N = 576	N = 562	N = 630	N = 508	
Large waist circumference	Incidence	225 (39.06%)	163 (29%)	150 (23.81%)	89 (17.52%)	<0.0001
	Odds ratio	1	0.637 (0.498–0.816)	0.487 (0.38–0.625)	0.331 (0.25–0.44)	<0.0001
High triglycerides	Incidence	160 (27.78%)	136 (24.2%)	116 (18.41%)	75 (14.76%)	<0.0001
	Odds ratio	1	0.83 (0.636–1.082)	0.587 (0.447–0.77)	0.45 (0.332–0.611)	<0.0001
Low HDL cholesterol	Incidence	280 (48.61%)	270 (48.04%)	294 (46.67%)	232 (45.67%)	0.7615
	Odds ratio	1	0.977 (0.775–1.233)	0.925 (0.738–1.16)	0.889 (0.7–1.129)	0.7619
High blood pressure	Incidence	203 (35.24%)	193 (34.34%)	174 (27.62%)	156 (30.71%)	0.0173
	Odds ratio	1	0.961 (0.753–1.227)	0.701 (0.549–0.895)	0.814 (0.631–1.05)	0.0176
High glucose	Incidence	126 (21.88%)	93 (16.55%)	94 (14.92%)	75 (14.76%)	0.0037
	Odds ratio	1	0.708 (0.526–0.953)	0.626 (0.466–0.841)	0.618 (0.451–0.847)	0.0039
Excluding heavy drinkers		N = 527	N = 528	N = 578	N = 466	
Large waist circumference	Incidence	209 (39.66%)	156 (29.55%)	136 (23.53%)	82 (17.6%)	<0.0001
	Odds ratio	1	0.638 (0.494–0.824)	0.468 (0.361–0.607)	0.325 (0.242–0.437)	<0.0001
High triglycerides	Incidence	138 (26.19%)	123 (23.3%)	105 (18.17%)	64 (13.73%)	<0.0001
	Odds ratio	1	0.856 (0.647–1.133)	0.626 (0.47–0.834)	0.449 (0.323–0.623)	<0.0001
Low HDL cholesterol	Incidence	264 (50.09%)	263 (49.81%)	279 (48.27%)	223 (47.85%)	0.8592
	Odds ratio	1	0.989 (0.777–1.259)	0.93 (0.734–1.177)	0.914 (0.712–1.173)	0.8593
High blood pressure	Incidence	182 (34.54%)	177 (33.52%)	158 (27.34%)	144 (30.9%)	0.0465
	Odds ratio	1	0.956 (0.741–1.233)	0.713 (0.552–0.921)	0.848 (0.65–1.106)	0.0471
High blood glucose	Incidence	112 (21.25%)	79 (14.96%)	80 (13.84%)	65 (13.95%)	0.0021
	Odds ratio	1	0.652 (0.475–0.895)	0.595 (0.434–0.816)	0.601 (0.43–0.84)	0.0023

Data are OR (95% CI) or frequency (%). *P* values show the trend. A *P* value <0.05 was considered statistically significant.

### The Additional Contribution of AST-to-ALT ratio to Predicting Risk of High Metabolic Syndrome

**Fig 2:**
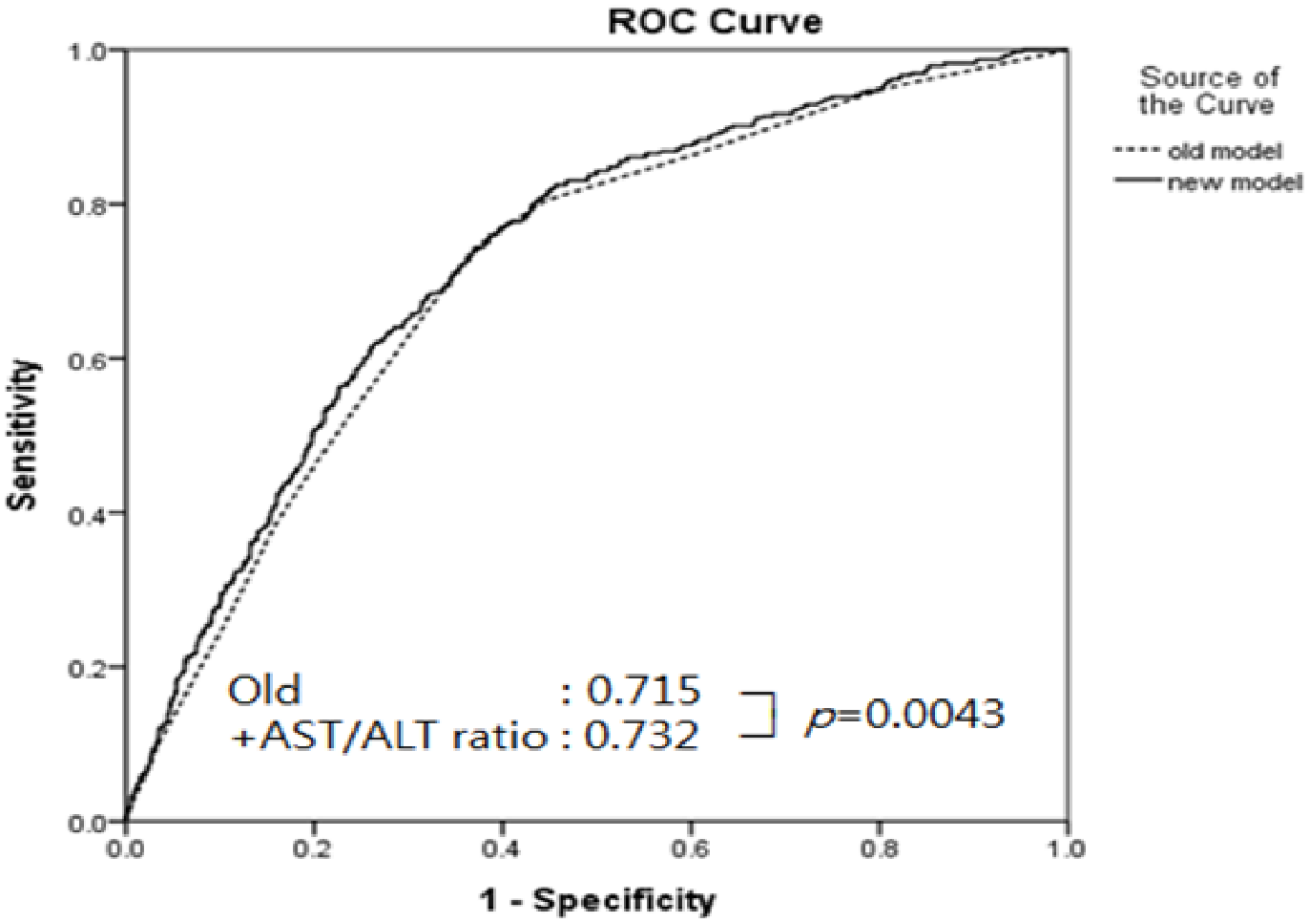
The additional contribution of the AST-to-ALT ratio to predicting the risk of metabolic syndrome.

The consistency observed in the association between the AST-to-ALT ratio and the risk of metabolic syndrome in the further analysis centered on all subjects in this cohort study. First, we measured the AUC of the AST-to-ALT ratio, ALT, GGT, and AST levels to predict the development of metabolic syndrome (Table E in S1 Tables). The AUCs for AST-to-ALT, ALT, and GGT were 0.611 (0.580–0.642), 0.604 (0.573–0.635), and 0.601(0.571–0.630), respectively. The baseline risk factors of metabolic syndrome and additional AST-to-ALT ratio model are shown in Table 4 to predict the future development of metabolic syndrome obtained with an AUC. The addition of the AST-to-ALT ratios to the models of individual components of metabolic syndrome significantly increased the AUCs. We also evaluated the prediction of new cases of metabolic syndrome by baseline variables of AST-to-ALT ratio over and above the information contributed by individual component of metabolic syndrome. The AUC predicts incident metabolic syndrome employing each component of metabolic syndrome (large waist circumference, high triglycerides, low HDL cholesterol, high blood pressure, and high blood glucose) to be 0.715 (0.688–0.741). After the AST-to-ALT ratio was added to this model, the resulting AUC was 0.732 (95% CI: 0.706–0.758, P = 0.0043) (Fig 2). We also calculated the additional predictive ability of other enzymes ALT, GGT, and AST to predict the future risk of developing metabolic syndrome beyond the information presented by different components of metabolic syndrome (Table F in S1 Tables).

**Table 4:** Comparison of AUC of each component of metabolic syndrome and additional AST-to-ALT ratio model.

	Each component’s AUC (95% CI)	AST/ALT additional model AUC (95%CI) AUC (95% CI)	p-value
All subjects			
Large waist circumference	0.569 (0.546–0.592)	0.641 (0.546–0.592)	<0.0001
High triglycerides	0.543 (0.521–0.564)	0.623 (0.591–0.654)	<0.0001
Low HDL cholesterol	0.537 (0.51–0.564)	0.615 (0.584–0.645)	<0.0001
High blood pressure	0.563 (0.536–0.59)	0.627 (0.597–0.657)	<0.0001
High blood glucose	0.549 (0.528–0.569)	0.639 (0.608–0.669)	<0.0001
5 components	0.715 (0.688–0.741)	0.732 (0.706–0.758)	0.0043

AUC, area under the ROC curve. *P* value is for the comparison of AUC between the model with each component of metabolic syndrome and the model with the addition of the AST-to-ALT ratio.

To measure the ability of the baseline AST-to-ALT ratio to predict the future onset of metabolic syndrome, we also calculated the ideal cut-off value of the AST-to-ALT ratio to define a discretional component of metabolic syndrome by the Youden index, which was 1.15 (data not shown). The NRI and IDI for prediction models including AST-to-ALT ratio were 0.23 (95% CI: 0.124–0.337, P<0.0001) and 0.0094 (95% CI: 0.0046–0.0143, p<0.0001).

## Discussion

In this longitudinal cohort study, we observed that the serum AST-to-ALT ratio was an independent negative predictor of the onset of metabolic syndrome and its individual components, except for HDL cholesterol, in the general Korean population. This independent relationship between the AST-to-ALT ratio and metabolic syndrome was not gender-biased and was unaffected by the exclusion of heavy drinkers. Moreover, our study showed that the serum AST-to-ALT ratio may improve the predictive power to accurately identify participants with risk for incident metabolic syndrome, beyond the information contributed by each of its components.

The serum AST-to-ALT ratio serves as a proxy measure for NAFLD and was shown to be inversely associated with metabolic syndrome and insulin resistance in clinical and epidemiological studies [9, 21, 22]. Despite strong evidence about the association between the AST-to-ALT ratio and obesity-related metabolic disorders, there have been little data from prospective studies based on the incremental predictive value of the serum AST-to-ALT ratio for the onset of metabolic syndrome [10, 23]. In our study, an increasing AST-to-ALT ratio was correlated with a consistent reduction in the onset of metabolic syndrome and its components. The prospective design, dose-dependent relationship and robustness of the association imply that the AST-to-ALT ratio may play a major role in the future diagnosis of metabolic syndrome.

We found that AUC was improved in the model in which the AST-to-ALT ratio was added to the metabolic syndrome components (0.715 to 0.732, P<0.0043). This indicates that the AST-to-ALT ratio enables the identification of incident metabolic syndrome independent of conventional risk assessment. We also used NRI and IDI to evaluate the prediction performance when the new biomarker (AST-to-ALT ratio) was added to a conventional metabolic syndrome risk model. The category-free NRI was 23% in our prospective cohort, which means that 23% of individuals in our study population were classified in the correct direction. Indeed, both NRI and IDI are more sensitive than AUC for stabilizing improvement in the predictive value [14, 24]. Improvements in the NRI, IDI, and c-statistics revealed that the AST-to-ALT ratio could have clinical importance in screening for the risk of metabolic syndrome, beyond the information suggested by traditional risk factors.

The relationship between metabolic syndrome and NAFLD or liver enzyme levels has been well established by liver biopsies, which are considered the gold standard for diagnosing NAFLD, although we did not use liver biopsies to confirm NAFLD-associated liver damage. Still, AST and ALT have been used as noninvasive surrogate markers of liver damage in epidemiological studies [25, 26] and the serum AST-to-ALT ratio is independently associated with metabolic syndrome and its components, consistent with the results of other studies in people of different ethnic origins [23, 27]. Moreover, some studies have reported that a possible mechanism for the association of the AST-to-ALT ratio with metabolic syndrome could be increased hepatic fat content [28], which adversely affects each component of metabolic syndrome. Another mechanism may involve an inflammatory effect in the liver that impairs insulin signaling, leading to a failure to suppress glucose production, and ultimately hyperglycemia [29–31]. Indeed, our results also indicate that the quartiles of AST-to-ALT ratios significantly predict hyperglycemia.

Recently, one study proposed that the liver enzyme ratio is the best surrogate marker of insulin resistance among non-obese Japanese adults [32]. Our study agrees with studies of particular adjusted models in the Korean and Chinese populations, wherein the liver enzyme ALT was significantly associated with metabolic syndrome, independent of insulin resistance measured by HOMA-IR [33, 34]. However, the above studies were focused on ALT (not AST), so we cannot confirm whether a similar relationship would exist with AST. Here, we used the HOMA index to estimate insulin resistance rather than the hyperinsulinemic-euglycemic clamp, which is invasive and requires a prolonged testing time, although many clinical trials have reported a good correlation between the two procedures for the assessment of insulin resistance [35, 36]. Additionally, we observed an independent association of the AST-to-ALT ratio and new onset of metabolic syndrome after adjustment for the potential inflammatory marker, hs-CRP. A possible explanation for the above result is the interaction between insulin resistance and hs-CRP. Insulin resistance is known to be involved with chronic inflammation, which is distinguished by elevated cytokine release and the activation of pro-inflammatory pathways [37, 38], indicating that insulin resistance may precede elevated hs-CRP by attenuating insulin-induced suppression of hepatic acute-phase plasma protein [39]. The exact mechanism by which insulin resistance, hs-CRP, and metabolic syndrome are related is still not clear in epidemiological studies. The above results and further adjustments suggest that the AST-to-ALT ratio has subsidiary physiopathology, distinct from other established risk factors. Our study clearly defines this uniqueness of the NAFLD marker, the AST-to-ALT ratio, as a prerequisite for diagnosing metabolic syndrome, but further verification is needed from long-term epidemiological studies.

Our study has several limitations. First, this study included middle-aged and elderly people living in rural settings with proportionately higher cases of metabolic syndrome [14]. Nonetheless, our cohort observed a similar pattern in the prevalence of metabolic syndrome to that of the Korean National Health and Nutrition Examination Survey (KNHANES) [12, 40, 41]. Moreover, the lifestyle change in Korea towards the Western pattern seems to be the primary cause for the increasing risk of metabolic syndrome. The time period of our study denoted a rapid increase in the prevalence rate of metabolic syndrome; therefore, it may not be extrapolated to other ethnic populations. Second, our study follow-up period was short, and thus we could not evaluate whether the association between the AST-to-ALT ratio and the onset of metabolic syndrome would endure long-term. Third, we could not eliminate the probability of confounding influences by viral liver disease. However, in previous reports from the third KNHANES, the prevalence of hepatitis B virus (HBV) infection among Korean adults over 40 years of age was 4% (4.2% for men and 3.8% for women [42]). Therefore, this could not have been a major confounder in our study population. Finally, a single assessment of serum AST and ALT is not adequate to examine the extent of liver inflammation, and thus we may have underemphasized the strength of the association.

In conclusion, the results of this study confirm the existence of an independent relationship between the AST-to-ALT ratio (a liver injury marker) and incident metabolic syndrome, as well as its components, in a prospective cohort study. Our findings suggest that the AST-to-ALT ratio should be considered clinically important for the evaluation of future risk of developing metabolic syndrome. This marker may be a useful tool with which clinicians can stratify cardiometabolic risk in population-based studies.

## Supporting Information

S1 Dataset All relevant data are available in the paper and its Supporting Information files. (XLSX)

S1 Tables Table A. Baseline characteristics of the study subjects stratified by metabolic syndrome, and evaluation of the AST-to-ALT ratio in relation to the number of individual components of metabolic syndrome at baseline; Table B. Odds ratio and 95% confidence interval (CI) for the prevalence of metabolic syndrome according to the different quartiles of AST-to-ALT ratio; Table C. Odds ratio and 95% confidence interval (CI) for new-onset metabolic syndrome according to serial change quartiles of AST-to-ALT ratios; Table D. Odds ratio and 95% confidence interval (CI) for new-onset of metabolic syndrome according to different quartiles of ALT and GGT levels; Table E. The AUC curve of AST-to-ALT ratio, ALT, GGT, and AST levels to predict the development of metabolic syndrome; Table F. Comparison of the AUC curves for 5 components and additional predictive ability of the AST-to-ALT ratio, ALT, GGT, and AST for the future risk of metabolic syndrome.
(DOCX)
