# Supplementary material for: Incremental Predictive Value of Serum AST-to-ALT Ratio for Incident Metabolic Syndrome: The ARIRANG Study
Source: PLoS One. 2016 Aug 25;11(8):e0161304. doi: 10.1371/journal.pone.0161304 (PMC4999188; doi:10.1371/journal.pone.0161304)
Supplement: S1 Tables — Table A. Baseline characteristics of the study subjects stratified by metabolic syndrome, and evaluation of the AST-to-ALT ratio in relation to the number of individual components of metabolic syndrome at baseline; Table B. Odds ratio and 95% confidence interval (CI) for the prevalence of metabolic syndrome according to the different quartiles of AST-to-ALT ratio; Table C. Odds ratio and 95% confidence interval (CI) for new-onset metabolic syndrome according to serial change quartiles of AST-to-ALT ratios; Table D. Odds ratio and 95% confidence interval (CI) for new-onset of metabolic syndrome according to different quartiles of ALT and GGT levels; Table E. The AUC curve of AST-to-ALT ratio, ALT, GGT, and AST levels to predict the development of metabolic syndrome; Table F. Comparison of the AUC curves for 5 components and additional predictive ability of the AST-to-ALT ratio, ALT, GGT, and AST for the future risk of metabolic syndrome. (DOCX) [file pone.0161304.s002.docx]

**Table A. Baseline characteristics of the studied subjects stratified by metabolic syndrome, and evaluation of the AST-to-ALT ratio in relation to the number of individual components of metabolic syndrome at baseline.**

| **Variables** | | **All subjects** | | | **Excluding heavy drinkers** | | |
| --- | --- | --- | --- | --- | --- | --- | --- |
|  |  | **Metabolic syndrome**  **(n=1494)** | **Non-metabolic syndrome**  **(n=2276)** | **p-value** | **Metabolic syndrome**  **(n=1352)** | **Non-metabolic syndrome**  **(n=2099)** | **p-value** |
| Age (y) | | 56.68 ± 7.98 | 54.17 ± 8.20 | <.0001 | 56.86 ± 7.91 | 54.13 ± 8.17 | <0.0001 |
| Gender | Men | 664 (42.37%) | 903 (57.63%) | 0.0037 | 529 (41.43%) | 748 (58.57%) | 0.0381 |
|  | Women | 830 (37.68%) | 1373 (62.32%) |  | 823 (37.86%) | 1351 (62.14%) |  |
| Waist circumference (cm) | | 89.03 ± 7.29 | 80.09 ± 7.80 | <.0001 | 88.76 ± 7.28 | 79.83 ± 7.83 | <0.0001 |
| BMI (kg/m^2^) | | 26.36 ± 2.93 | 23.52 ± 2.72 | <.0001 | 26.38 ± 2.94 | 23.53 ± 2.75 | <0.0001 |
| Fasting glucose (mg/dL) | | 104.5 ± 25.61 | 91.12 ± 13.89 | <.0001 | 103.8 ± 25.23 | 90.92 ± 14.14 | <0.0001 |
| SBP (mmHg) | | 138.6 ± 17.58 | 125.5 ± 17.20 | <.0001 | 138.2 ± 17.60 | 125.3 ± 17.16 | <0.0001 |
| DBP (mmHg) | | 87.36 ± 10.91 | 80.03 ± 11.30 | <.0001 | 86.89 ± 10.78 | 79.81 ± 11.25 | <0.0001 |
| HDL cholesterol (mg/dL) | | 40.80 ± 8.58 | 49.35 ± 11.00 | <.0001 | 40.68 ± 8.40 | 49.05 ± 10.62 | <0.0001 |
| LDL cholesterol (mg/dL) | | 121.3 ± 34.65 | 115.7 ± 30.66 | <.0001 | 122.8 ± 34.44 | 116.3 ± 30.65 | <0.0001 |
| Triglyceride (mg/dL) | | 207.2 ± 138.5 | 110.8 ± 59.31 | <.0001 | 200.1 ± 125.2 | 108.2 ± 54.95 | <0.0001 |
| hs-CRP (mg/L) | | 2.24 ± 4.63 | 1.82 ± 5.16 | 0.0086 | 2.27 ± 4.71 | 1.83 ± 5.28 | 0.0106 |
| HOMA-IR | | 2.65 ± 2.15 | 1.74 ± 0.97 | <.0001 | 2.64 ± 2.17 | 1.74 ± 0.97 | <0.0001 |
| **AST (units/L)** | | **30.35 ± 47.40** | **27.01 ± 14.42** | **0.0081** | **29.50 ± 48.83** | **26.34 ± 12.55** | **0.0201** |
| **ALT (units/L)** | | **29.81 ± 29.45** | **23.01 ± 15.58** | **<.0001** | **29.01 ± 30.02** | **22.44 ± 14.41** | **<0.0001** |
| **AST/ALT ratio** | | **1.11 ± 0.34** | **1.30 ± 0.42** | **<.0001** | **1.11 ± 0.33** | **1.30 ± 0.42** | **<0.0001** |
| Smoker (%) | | 448 (30.09%) | 638 (28.12%) | 0.1927 | 350 (25.98%) | 519 (24.81%) | 0.4390 |
| Exercise (%) | | 476 (32.03%) | 707 (31.17%) | 0.5794 | 434 (32.29%) | 651 (31.13%) | 0.4760 |
| Drinking (%) | | 710 (47.68%) | 1008 (44.37%) | 0.0458 | 568 (42.17%) | 831 (39.67%) | 0.1447 |
| **No of components** | | **AST/ALT ratio** |  |  | **AST/ALT ratio** |  |  |
| **0** | | **1.36 ± 0.44** |  | **<.0001** | **1.37 ± 0.45** |  | **<.0001** |
| **1** | | **1.35 ± 0.47** |  |  | **1.35 ± 0.47** |  |  |
| **2** | | **1.23 ± 0.36** |  |  | **1.23 ± 0.35** |  |  |
| **3** | | **1.15 ± 0.35** |  |  | **1.15 ± 0.35** |  |  |
| **≥4** | | **1.06 ± 0.31** |  |  | **1.06 ± 0.30** |  |  |

Data are expressed as mean ± standard deviation or number (%). BMI, Body mass index; SBP, Systolic blood pressure; DBP, Diastolic blood pressure; hs-CRP, high-sensitivity C-reactive protein; HOMA-IR, homeostasis model assessment-estimated insulin resistance; AST, aspartate aminotransferase; ALT, alanine aminotransferase

**Table B. Odds ratios and 95% confidence interval (CI) for prevalence of metabolic syndrome according to the different quartiles of AST to ALT ratio.**

|  | Q1 | Q2 | Q3 | Q4 | p-value |
| --- | --- | --- | --- | --- | --- |
| All subjects | N=943 | N=959 | N=937 | N=931 |  |
| AST-to-ALT ratio | ~ 0.96 | 0.96 ~ 1.18 | 1.18 ~ 1.42 | 1.42 ~ |  |
| prevalence | 552 (58.54%) | 415 (43.27%) | 299 (31.91%) | 228 (24.49%) | <0.0001 |
| Crude OR | 1 | 0.540 (0.450~0.648) | 0.332 (0.275~0.401) | 0.230 (0.189~0.280) | <0.0001 |
| Model 1 | 1 | 0.498 (0.413~0.600) | 0.288 (0.237~0.350) | 0.194 (0.158~0.238) | <0.0001 |
| Model 2 | 1 | 0.609 (0.495~0.750) | 0.404 (0.325~0.502) | 0.361 (0.287~0.453) | <0.0001 |
| Model 3 | 1 | 0.678 (0.547~0.839) | 0.500 (0.399~0.626) | 0.465 (0.367~0.589) | <0.0001 |
| Excluding heavy drinkers | N=863 | N=861 | N=876 | N=851 |  |
| AST-to-ALT ratio | ~ 0.96 | 0.96 ~ 1.18 | 1.18 ~ 1.42 | 1.42 ~ |  |
| prevalence | 502 (58.17%) | 370 (42.97%) | 276 (31.51%) | 204 (23.97%) | <0.0001 |
| Crude OR | 1 | 0.542 (0.448~0.656) | 0.331 (0.272~0.403) | 0.227 (0.184~0.279) | <0.0001 |
| Model 1 | 1 | 0.498 (0.41~0.606) | 0.287 (0.234~0.351) | 0.193 (0.156~0.239) | <0.0001 |
| Model 2 | 1 | 0.598 (0.481~0.744) | 0.394 (0.314~0.494) | 0.354 (0.279~0.450) | <0.0001 |
| Model 3 | 1 | 0.663 (0.530~0.830) | 0.491 (0.388~0.621) | 0.451 (0.352~0.579) | 0.0011 |

Data are OR (95% CI) or n (%). Model 1: adjusted for age. Model 2: Model 1, plus additional adjustment for baseline BMI, LDL cholesterol, smoking, regular exercise, and alcohol drinking. Model 3: Model 2, plus additional adjustment for baseline hs-CRP and HOMA-IR

**Table C. Odds ratios and 95% confidence interval (CI) for new-onset metabolic syndrome according to serial change quartiles of AST-to-ALT ratios.**

|  | Q1 | Q2 | Q3 | Q4 | p-value |
| --- | --- | --- | --- | --- | --- |
| All subjects | N=570 | N=568 | N=569 | N=569 |  |
| AST-to-ALT ratio | ~ -0.235 | -0.235 ~ -0.029 | -0.029 ~ 0.168 | 0.168 ~ |  |
| incidence | 98 (17.19%) | 113 (19.89%) | 100 (17.57%) | 84 (14.76%) | 0.1544 |
| Crude OR | 1 | 1.196 (0.886~1.614) | 1.027 (0.756~1.395) | 0.834 (0.756~1.395) | 0.1560 |
| Model 1 | 1 | 1.200 (0.889~1.621) | 1.026 (0.755~1.395) | 0.842 (0.613~1.158) | 0.1697 |
| Model 2 | 1 | 1.151 (0.842~1.575) | 0.934 (0.677~1.288) | 0.814 (0.583~1.136) | 0.2035 |
| Model 3 | 1 | 1.139 (0.832~1.560) | 0.915 (0.663~1.263) | 0.796 (0.570~1.113) | 0.1751 |
| Excluding heavy drinkers | N=524 | N=525 | N=531 | N=519 |  |
| AST-to-ALT ratio | ~ -0.238 | -0.238 ~ -0.032 | -0.032 ~ 0.167 | 0.167 ~ |  |
| incidence | 91 (17.37%) | 102 (19.43%) | 93 (17.51%) | 76 (14.64%) | 0.2360 |
| Crude OR | 1 | 1.147 (0.839~1.569) | 1.010 (0.735~1.389) | 0.816 (0.586~1.138) | 0.2378 |
| Model 1 | 1 | 1.154 (0.843~1.579) | 1.005 (0.731~1.383) | 0.826 (0.592~1.152) | 0.2564 |
| Model 2 | 1 | 1.094 (0.788~1.519) | 0.911 (0.652~1.273) | 0.779 (0.549~1.105) | 0.2510 |
| Model 3 | 1 | 1.083 (0.779~1.505) | 0.894 (0.639~1.251) | 0.759 (0.534~1.078) | 0.2058 |

Data are OR (95% CI) or n (%). Model 1: adjusted for age. Model 2: Model 1, plus additional adjustment for baseline BMI, LDL cholesterol, smoking, regular exercise, and alcohol drinking. Model 3: Model 2, plus additional adjustment for baseline hs-CRP and HOMA-IR

**Table D. Odds ratio and 95% confidence interval (CI) for new-onset of metabolic syndrome according to different quartiles of ALT and GGT levels.**

|  | Q1 | Q2 | Q3 | Q4 | p-value |
| --- | --- | --- | --- | --- | --- |
| All subjects |  |  |  |  |  |
| ALT | ~ 15 | 15-19 | 19-26 | 26~ |  |
| incidence | 68 (11.58%) | 78 (13.78%) | 117 (19.31%) | 132 (25.53%) | <0.0001 |
| Crude OR | 1 | 1.220 (0.861-1.727) | 1.826 (1.322-2.523) | 2.617 (1.898-3.607) | <0.0001 |
| Model 1 | 1 | 1.182 (0.834-1.676) | 1.759 (1.271-2.434) | 2.550 (1.848-3.517) | <0.0001 |
| Model 2 | 1 | 1.049 (0.732-1.502) | 1.389 (0.989-1.950) | 1.717 (1214-2.428) | 0.005 |
| Model 3 | 1 | 1.048 (0.732-1.502) | 1.368 (0.974-1.923) | 1.668 (1.178-2.362) | 0.0100 |
| GGT | ~ 11 | 11-17 | 17-28 | 28~ |  |
| incidence | 61 (10.02%) | 94 (15.82%) | 110 (21.36%) | 130 (23.30%) | <0.0001 |
| Crude OR | 1 | 1.689 (1.197-2.383) | 2.440 (1.740-3.422) | 2.729 (1.963-3.793) | <0.0001 |
| Model 1 | 1 | 1.631 (1.154-2.305) | 2.323 (1.652-3.267) | 2.603 (1.868-3.627) | <0.0001 |
| Model 2 | 1 | 1.367 (0.955-1.956) | 1.809 (1.253-2.613) | 1.952 (1.328-2.870) | 0.0031 |
| Model 3 | 1 | 1.377 (0.961-1.972) | 1.793(1.240-2.592) | 1.901 (1.290-2.800) | 0.0052 |
| Excluding heavy drinkers |  |  |  |  |  |
| ALT | ~ 15 | 15-19 | 19-25 | 25~ |  |
| incidence | 66 (11.64%) | 75 (13.99%) | 98 (20.08%) | 123 (24.21%) | <0.0001 |
| Crude OR | 1 | 1.235 (0.867-1.760) | 1.907 (1.359-2.677) | 2.425 (1.748-3.364) | <0.0001 |
| Model 1 | 1 | 1.195 (0.837-1.705) | 1.829 (1.301-2.572) | 2.364 (1.702-3.282) | <0.0001 |
| Model 2 | 1 | 1.036 (0.718-1.495) | 1.430 (1.001-2.042) | 1.526 (1.071-2.175) | 0.0326 |
| Model 3 | 1 | 1.036 (0.718-1.496) | 1.400 (0.979-2.003) | 1.483 (1.039-2.117) | 0.0563 |
| GGT | ~ 11 | 11-16 | 16-25 | 25~ |  |
| incidence | 61 (10.10%) | 78 (16.46%) | 104 (20.84%) | 119 (22.80%) | <0.0001 |
| Crude OR | 1 | 1.753 (1.224-2.511) | 2.344 (1.665-3.298) | 2.628 (1.881-3.672) | <0.0001 |
| Model 1 | 1 | 1.692 (1.180-2.427) | 2.249 (1.595-3.171) | 2.505 (1.788-3.509) | <0.0001 |
| Model 2 | 1 | 1.440 (0.991-2.092) | 1.672 (1.158-2.413) | 1.694 (1.151-2.494) | 0.0295 |
| Model 3 | 1 | 1.456 (1.001-2.118) | 1.655 (1.146-2.392) | 1.634 (1.107-2.412) | 0.0412 |

Data are OR (95% CI) or n (%). Model 1: adjusted for age. Model 2: Model 1, plus additional adjustment for baseline BMI, LDL cholesterol, smoking, regular exercise, and alcohol drinking. Model 3: Model 2, plus additional adjustment for baseline hs-CRP and HOMA-IR

**Table E. The AUCs of AST to ALT ratio, ALT, GGT, and AST levels to predict the development of metabolic syndrome.**

| Variable | AUC (95% CI) | p-value | p-value |
| --- | --- | --- | --- |
| AST/ALT | 0.611 (0.580-0.642) | 0.0003 | 0.5555 |
| ALT | 0.604 (0.573-0.635) | <0.0001 | 0.8356 |
| GGT | 0.601 (0.571-0.630) | <0.0001 | - |
| AST | 0.537 (0.506-0.568) | - |  |

AUC, area under the ROC curve. *P* value is for the comparison of AUC between the model with AST-to-ALT ratio, ALT, GGT, and AST for predicting new onset of metabolic syndrome.

**Table F.** **Comparison of AUCs for 5 components and additional predictive ability of AST to ALT ratio, ALT, GGT, AST for future risk of metabolic syndrome.**

| Additional variable | AUC (95% CI) | p-value |
| --- | --- | --- |
| 5 components | 0.715 (0.688-0.741) | - |
| AST/ALT | 0.732 (0.706-0.758) | 0.0043 |
| ALT | 0.729 (0.703-0.755) | 0.0028 |
| GGT | 0.722 (0.696-0.748) | 0.0093 |
| AST | 0.718 (0.691-0.744) | 0.388 |

AUC, area under the ROC curve. *P* value is for the comparison of AUC between the model with components of metabolic syndrome and the model with the addition of the AST-to-ALT ratio, ALT, GGT, and AST.
